# Genetic diversity and phylogenetic relationship of nine sheep populations based on microsatellite markers

**DOI:** 10.5194/aab-64-7-2021

**Published:** 2021-01-06

**Authors:** Qing Xia, Xiangyu Wang, Zhangyuan Pan, Rensen Zhang, Caihong Wei, Mingxing Chu, Ran Di

**Affiliations:** Key Laboratory of Animal Genetics and Breeding and Reproduction of the Ministry of Agriculture and Rural Affairs, Institute of Animal Science, Chinese Academy of Agricultural Sciences, Beijing 100193, PR China

## Abstract

The objective of this study was to assess the genetic diversity and phylogenetic relationship of nine sheep populations, including two famous high prolific populations and seven popular mutton populations raised in China. Overall, these sheep populations in this study exhibited a rich genetic diversity. Both the expected heterozygosity and Nei's unbiased gene diversity ranged from 0.64 to 0.75, with the lowest value found in Dorset sheep (DST) and the highest in Hu sheep (HUS) and Ba Han sheep (BAS). The polymorphic information content (PIC) varied between 0.59 in DST and 0.71 in HUS and BAS. Specifically, for individual breeds, the small-tail Han sheep (STH) and the four introduced populations did not display the expected diversity; therefore more attention should be paid to the maintenance of diversity during management of these populations. The results of un-weighted pair-group method (UPGMA) phylogenetic tree and structure analysis indicated that the nine investigated populations can be divided into two groups. Suffolk (SUF) and DST were clustered in one group, and the other group can be further divided into three clusters: German Mutton Merino (GMM)–BAS–Bamei Mutton sheep (BAM), HUS–STH and Du Han (DOS)–Dorper (DOP). This clustering result is consistent with sheep breeding history. TreeMix analysis also hinted at the possible gene flow from GMM to SUF. Together, an in-depth view of genetic diversity and genetic relationship will have important implications for breed-specific management.

## 1 Background

Sheep is one of the earliest domesticated animal species (Zeder et al., 2006; Bernardo et al., 2009). It provides meat and milk with high-quality protein and useful accessory products (wool and skin) for human. Therefore, sheep became an important livestock species and promoted the spread of human farming civilization (Jing and Zhang, 2009). China has a long history of sheep domestication (Kawamura et al., 2005) and famous sheep breeds with high reproductive performance. For example, small-tail Han sheep (STH) and Hu sheep (HUS) had attracted much attention for their excellent characteristics of high litter size and year-round estrus (China National Commission of Animal Genetic Resources, 2011). In addition to reproductive traits, meat production traits are also the focus of attention for sheep farmers. Since the growth rate and performance of meat production in Chinese sheep populations are relatively low compared with famous mutton sheep breeds worldwide (e.g., Dorper (DOP), Suffolk (SUF), Dorset (DST) and German Mutton Merino (GMM)), these perfect mutton breeds were introduced to China in the past two decades and are now common in many farms of China. However, with the introduction of foreign breeds to improve production performance of indigenous purebred sheep, the number of indigenous purebred sheep with high fertility is gradually decreasing (especially for STH) in China. Therefore, the evaluation of current genetic diversity situation in these sheep populations is very essential for breed protection. In addition, this study also involved seven mutton sheep populations raised in China including four introduced mutton populations mentioned above and three mutton populations in Inner Mongolia of China, which is the main producing area of mutton in China. The genetic diversity assessment for the seven mutton populations will be helpful for the formulation of plans of further management and utilization for these populations.

Microsatellite markers belong to a class of neutral markers, which have rich polymorphism in population and are widely distributed in the genome; therefore they are suitable for genetic diversity analysis. Previous studies indicated that microsatellite markers had been well used to assess genetic diversity and population structure of sheep breeds (Di et al., 2012; Dotsev et al., 2018; Madilindi et al., 2019; Vargas et al., 2018; Laoun et al., 2020; Ullah et al., 2020) and goat breeds (Carvalho et al., 2015; Câmara et al., 2017; E et al., 2019; Liu et al., 2019). Therefore, in this study, a panel of 26 microsatellite markers was employed to evaluate the genetic diversity, population structure and phylogenetic relationship of the nine sheep populations. The results will provide important information for the implementation of breed-specific management, propagation or conservation programs.

**Table 1:** Summary information of nine sheep populations in this study.

Population	Population	Sample number		Country of	Introduced and sampling location
	code	female	male	origin	
Small-tail Han sheep	STH	30	30	China	Shandong Province, China
Hu sheep	HUS	30	30	China	Zhejiang Province, China
Bamei Mutton sheep	BAM	30	30	China	Bayannur, Inner Mongolia, China
Ba Han sheep	BAS	30	30	China	Bayannur, Inner Mongolia, China
Du Han sheep	DOS	30	30	China	Bayannur, Inner Mongolia, China
Suffolk	SUF	30	30	Britain	Fuchuan Breeding Technology Co., Ltd, Inner Mongolia, China
Dorset	DST	30	30	Britain	Fuchuan Breeding Technology Co., Ltd, Inner Mongolia, China
Dorper	DOP	30	30	South Africa	Fuchuan Breeding Technology Co., Ltd, Inner Mongolia, China
German Mutton Merino	GMM	30	30	Germany	Fuchuan Breeding Technology Co., Ltd, Inner Mongolia, China

## 2 Material and methods

### 2.1 Animal ethics

All experiments involving animals were authorized by the Animal Ethics Committee of the Institute of Animal Science, Chinese Academy of Agricultural Sciences (no. IAS2019-49).

### 2.2 Animals and DNA extraction

Nine sheep populations – small-tail Han (STH), Hu (HUS), Suffolk (SUF), Dorset (DST), Dorper (DOP), German Mutton Merino (GMM), Bamei Mutton (BAM), Ba Han (BAS) and Du Han (DOS) – were analyzed in this study (see Table 1 for specific sample information). A total of 5 mL jugular vein blood was collected from 60 individuals (30 ewes and 30 rams) in each population. Then genomic DNA was extracted according to protocol described by Russell (Russell and Sambrook, 2001). The DNA was dissolved in TE buffer (10 mmol/L Tris-HCL (pH 8.0), 1 mmol/L EDTA (pH 8.0)) and stored at -80${}^{\circ }$C in a freezer.

**Table 2:** Information of amplification and genetic parameter statistics for each microsatellite locus.

Loci	Chromosome	Primer	Annealing	Fragment	Number	$N_{\text{E}}$	Rt	PIC	$F_{\text{IT}}$	$F_{\text{IS}}$	$F_{\text{ST}}$
		sequence (5${}^{\prime }$–3${}^{\prime }$)	temperature (${}^{\circ }$)	size	of alleles						
OARFCB193	11	F: TTCATCTCAGACTGGGATTCAGAAAGGC R: GCTTGGAAATAACCCTCCTGCATCCC	54	94–136	21	3.1965	9.7089	0.5759	0.1596	0.0400	0.1246
OARJMP29	24	F: GTATACACGTGGACACCGCTTTGTAC R: GAAGTGGCAAGATTCAGAGGGGAAG	56	93–159	23	4.7553	10.4248	0.7111	0.1334	0.0750	0.0631
OARJMP58	26	F: GAAGTCATTGAGGGGTCGCTAACC R: CTTCATGTTCACAGGACTTTCTCTG	58	125–173	20	4.9553	10.2537	0.7124	0.0299	-0.0438	0.0707
OARFCB304	19	F: CCCTAGGAGCTTTCAATAAAGAATCGG R: CGCTGCTCTCAACTGGGTCAGGG	56	150–188	21	4.7104	11.3268	0.7046	0.0568	-0.0105	0.0666
BM8125	17	F: CTCTATCTGTGGAAAAGGTGGG R: GGGGGTTAGACTTCAACATACG	50	104–122	9	2.3668	6.0283	0.4894	0.1198	0.0265	0.0958
OARFCB128	2	F: ATTAAAGCATCTTCTCTTTATTTCCTCGC R: CAGCTGAGCAACTAAGACATACATGCG	55	96–132	15	5.6435	9.1417	0.7229	0.1022	0.0212	0.0827
OARVH72	25	F: CTCTAGAGGATCTGGAATGCAAAGCTC R: GGCCTCTCAAGGGGCAAGAGCAGG	57	119–139	10	4.6513	7.4639	0.6425	0.1040	-0.0299	0.1300
OARHH47	18	F: TTTATTGACAAACTCTCTTCCTAACTCCACC R: GTAGTTATTTAAAAAAATATCATACCTCTTAAGG	58	121–149	15	6.9942	10.4087	0.7823	0.0838	0.0272	0.0583
DYMS1	20	F: AACAACATCAAACAGTAAGAG R: CATAGTAACAGATCTTCCTACA	59	161–209	20	9.2113	13.2710	0.8126	0.1393	0.0752	0.0694
SRCRSP1	13	F: TGCAAGAAGTTT TTCCAGAGC R: ACCCTGGTTTCACAA AAG G	54	113–143	13	3.1794	5.9478	0.5929	0.0557	-0.0012	0.0568
SRCRSP5	18	F: GGACTCTACCAACTGAGCTACAAG R: GTT TCTTTG AAATGAAGCTAAAGCAATGC	56	145–159	8	2.6112	4.6968	0.4761	0.4481	0.3572	0.1414
SRCRSP9	12	F: AGAGGATCTGGA AATGGAATC R: GCACTCTTTTCAGCCCTAATG	55	100–136	16	2.7415	5.8956	0.5208	0.0554	-0.0500	0.1004
MCM140	6	F: GTTCGTACTTCTGGGTACTGGTCTC R: GTCCATGGATTTGCAGAGTCAG	60	173–197	13	4.6543	9.5181	0.7070	0.0281${}^{*}$	-0.0345	0.0605
MAF33	9	F: GATCTTTGTTTCAATCTATTCCAATTTC R: GATCATCTGAGTGTGAGTATATACAG	60	117–145	15	4.3296	8.4649	0.6764	0.0698	0.0002	0.0696
MAF209	17	F: GATCACAAAAAGTTGGATACAACCGTGG R: TCATGCACTTAAGTATGTAGGATGCTG	63	99–131	15	7.7184	10.4243	0.8079	0.1028	0.0582	0.0473${}^{*}$
INRA063	14	F: ATTTGCACAAGCTAAATCTAACC R: AAACCACAGAAATGCTTGGAAG	58	147–199	23	8.0805	12.4833	0.7823	0.1226	0.0421	0.0840
MAF214	16	F: GGGTGATCTTAGGGAGGTTTTGGAGG R: AATGCAGGAGATCTGAGGCAGGGACG	58	163–257	25	3.0660	6.9890	0.5735	0.1111	0.0334	0.0804
ILSTS11	9	F: GCTTGCTACATGGAAAGTGC R: CTAAAATGCAGAGCCCTACC	55	266–286	11	4.0005	6.9549	0.6282	0.1035	-0.0047	0.1077
MCM527	5	F: GTCCATTGCCTCAAATCAATTC R: AAACCACTTGACTACTCCCCAA	58	160–186	12	5.0537	6.7644	0.6959	0.0799	0.0023	0.0778
OARFCB226	2	F: CTATATGTTGCCTTTCCCTTCCTGC R: GTGAGTCCCATAGAGCATAAGCTC	60	104–158	22	5.0904	11.7429	0.7221	0.0466	-0.0271	0.0717
ILSTS28	3	F: TCCAGATTTTGTACCAGACC R: GTCATGTCATACCTTTGAGC	53	124–172	19	4.5920	10.2470	0.7047	0.0517	-0.0067	0.0580
MAF70	4	F: CACGGAGTCACAAAGAGTCAGACC R: GCAGGACTCTACGGGGCCTTTGC	60	116–164	21	9.0975	14.0685	0.8325	0.0342	-0.0121	0.0458${}^{*}$
HUJ616	13	F: TTCAAACTACACATTGACAGGG R: GGACCTTTGGCAATGGAAGG	54	114–174	24	3.8767	9.8103	0.6521	0.0482	-0.0363	0.0816
TGLA53	12	F: GCTTTCAGAAATAGTTTGCATTCA R: ATCTTCACATGATATTACAGCAGA	55	126–160	17	5.9463	10.2072	0.7557	0.2073	0.1551	0.0618
MCMA54	21	F: AACGTCCATCAGTAGATGAATGG R: GTAGCATGATGTCTTGGCCACT	63	148–160	13	2.3860	5.2113	0.4754	0.1440	0.0541	0.0950
MCM159	15	F: GATGGTCTTGTTTCTGAATCATTGA R: TCAGACAGGACTAAAGCGACTTACA	60	125–159	14	3.1636	7.0872	0.5843	0.1304	0.0363	0.0976
Mean						4.8489	9.0208	0.6671	0.1033	0.0264	0.0790

$N_{\text{E}}$: the effective number of alleles; Rt: the allelic richness over all samples per locus; $F_{\text{IT}}$: inbreeding coefficient; $F_{\text{IS}}$: fixation index within populations; $F_{\text{ST}}$: coefficient of relatedness between individuals from different populations; PIC: the polymorphic information content; Rt: the allelic richness over all samples per locus. ${}^{*}$ indicates a significant difference of locus in populations (P<0.05).

### 2.3 PCR amplification and genotyping

All sheep were genotyped using 26 microsatellite markers distributed in different chromosomes. Information of 26 microsatellite markers is summarized in Table 2. The primers for the first 24 microsatellite markers were recommended by the joint ISAG–FAO Standards Committee for analysis of sheep genetic diversity (Arranz et al., 1998, 2001; Martin-Burriel et al., 1999; Diez-Tascon et al., 2000; Stahlberger-Saitbekova et al., 2001; Pariset et al., 2003; Tapio et al., 2003). The primers for the other two loci are selected from the report of Davies et al. (1995) and Di et al. (2012). Forward primers were 5${}^{\prime }$-labeled with fluorescent dyes (FAM or HEX, Sangon Biotech (Shanghai) Co., Ltd.). The PCR protocol refers to the report of Zhong et al. (2011). PCR amplifications were performed in 12 µL reaction volumes containing 50 ng of genomic DNA, 2.5 mM MgCl${}_{2}$, 250 µM of each dNTP, 0.025 µM of each primer, 1.25 units of *Taq* polymerase and 1 × magnesium-free PCR buffer (Takara, Japan). Amplifications were carried out using the GENEAMP PCR 9700 thermocycler (Applied Biosystems), with the following cycling parameters: 94 ${}^{\circ }$C for 5 min followed by 30 cycles of 94 ${}^{\circ }$C for 30 s, annealing at 55–60 ${}^{\circ }$C for 30 s, 72 ${}^{\circ }$C for 30 s and a final step at 72 ${}^{\circ }$C for 10 min. PCR products were diluted to 1/2–1/4 concentrations, and 0.75 µL of each diluted product was then mixed with an internal standard (Gene Scan 500 LIZ™, Applied Bio systems, USA) according to the manufacturer's instructions. Genotyping was performed using a Genetic Analyzer 3130 xl (Applied Bio systems, USA). Fragment analysis was performed using GENEMAPPER V3.7 software (Applied Biosystems). The third-order least squares method was used for allele size determination (Mburu et al., 2003). Genotyping was repeated once if individual samples failed to amplify.

### 2.4 Statistical analysis

The genetic diversity parameters of nine sheep populations, including mean number of effective alleles ($N_{\text{A}}$), the expected heterozygosity ($H_{\text{E}}$), the observed heterozygosity ($H_{\text{O}}$), the polymorphic information content (PIC) and Nei's unbiased gene diversity ($H_{\text{S}}$), were calculated using Microsatellite Toolkit software 3.1.1 (Park, 2008). The genetic parameters for each microsatellite locus, including the effective number of alleles ($N_{\text{E}}$), the allelic richness over all samples per locus (Rt), the fixation index within populations ($F_{\text{IS}}$), the fixation indices of total population ($F_{\text{IT}}$) and the pairwise differences between the populations ($F_{\text{ST}}$), were obtained using FSTAT 2.9.3.2 (Goudet et al., 2002). Dispan was used to calculate the genetic distance of $D_{\text{A}}$ and $D_{\text{S}}$ between populations and build the phylogenetic tree (Nei et al., 1983). Population structure was analyzed by Structure 2.3.4 with Bayesian clustering model, and its results were visualized by Distruct 1.1 (Falush et al., 2003; Pritchard et al., 2000; Rosenberg, 2003). TreeMix 1.13 was used to construct a maximum likelihood tree and infer migration events between branches (Pickrell et al., 2012).

**Table 3:** Genetic diversity information in the nine sheep populations.

Population	$N_{\text{A}}$	$H_{\text{E}}$	$H_{\text{O}}$	PIC	$H_{\text{S}}$
STH	10.50	0.73	0.69	0.69	0.73
HUS	10.08	0.75	0.71	0.71	0.75
SUF	8.12	0.67	0.63	0.63	0.67
DST	8.15	0.64	0.63	0.59	0.64
DOP	9.38	0.68	0.64	0.64	0.68
GMM	10.00	0.74	0.70	0.70	0.74
BAM	8.42	0.68	0.68	0.64	0.68
BAS	10.12	0.75	0.74	0.71	0.75
DOS	9.23	0.73	0.73	0.69	0.73
Mean	9.33	0.71	0.68	0.67	0.71

$N_{\text{A}}$: mean number of effective alleles; $H_{\text{E}}$: expected heterozygosity; $H_{\text{O}}$: observed heterozygosity; PIC: polymorphism information content; $H_{\text{S}}$: Nei's unbiased gene diversity.

## 3 Results

### 3.1 Genetic diversity in nine sheep populations

In this study, the genetic diversity analysis was firstly performed in nine sheep populations. The mean number of effective alleles ($N_{\text{A}}$) is the largest in STH (10.50) and the least in SUF (8.12). The expected heterozygosity ($H_{\text{E}}$) and observed heterozygosity ($H_{\text{O}}$) within the population ranged from 0.64 to 0.75 and 0.63 to 0.74, respectively, with the lowest values found in the DST and the highest in the BAS. The highest PIC was observed in HUS and BAS (0.71), followed by GMM (0.70), and the lowest value was observed in DST (0.59). The Nei's unbiased gene diversity ($H_{\text{S}}$) varied between 0.64 in DST and 0.75 in HUS and BAS (Table 3). Overall, these sheep populations in this study exhibited a rich genetic diversity. However, specifically for a single breed, the STH did not display the expected diversity. In addition, compared with other populations, the diversity of the four introduced populations raised in China was relatively low (especially in DST) in the nine sheep populations. Therefore, the above five populations (STH, DST, DOP, SUF, and GMM) exert more of our attention to the maintenance of diversity during population management.

**Figure 1:**
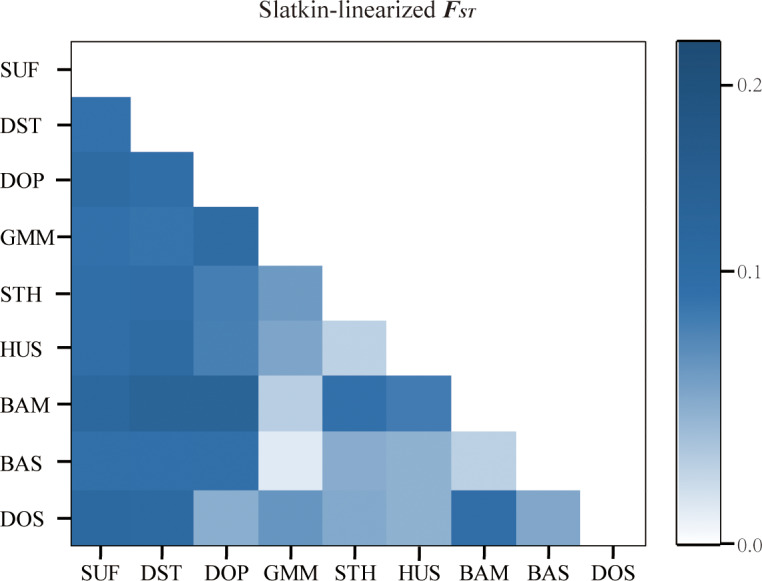
Pairwise differences among nine sheep populations ($F_{\text{ST}}$) using microsatellite markers.

Meanwhile, it was observed that all the microsatellite loci were polymorphic across nine populations. A total of 435 alleles were identified in nine sheep populations, and the number of alleles per locus ranged from 8 (SRCRSP5) to 25 (MAF214) (Table 2). The highest effective number of alleles ($N_{\text{E}}$) was observed at locus DYMS1 (9.2113) and the lowest at BM8125 (2.3668). Allelic richness over all samples per locus (Rt) was measured between 4.6968 (SRCRSP5) and 14.0685 (MAF70), with a mean of 9.0208. The PIC across all the populations ranged from 0.4754 (MCMA54) to 0.8325 (MAF70). In total, these loci were polymorphic and suitable for sheep genetic diversity analysis.

**Table 4:** Pairwise differences between population ($F_{\text{ST}}$) using microsatellite markers.

	STH	HUS	SUF	DST	DOP	GMM	BAM	BAS	DOS
STH	0.0000								
HUS	0.0302${}^{*}$	0.0000							
SUF	0.0954${}^{*}$	0.0974${}^{*}$	0.0000						
DST	0.1001${}^{*}$	0.1044${}^{*}$	0.0909${}^{*}$	0.0000					
DOP	0.0822${}^{*}$	0.0811${}^{*}$	0.1055${}^{*}$	0.096${}^{*}$	0.0000				
GMM	0.0648${}^{*}$	0.0583${}^{*}$	0.0916${}^{*}$	0.0891${}^{*}$	0.1025${}^{*}$	0.0000			
BAM	0.0911${}^{*}$	0.0841${}^{*}$	0.1124${}^{*}$	0.1212${}^{*}$	0.1212${}^{*}$	0.0321${}^{*}$	0.0000		
BAS	0.0536${}^{*}$	0.0501${}^{*}$	0.0937${}^{*}$	0.0936${}^{*}$	0.095${}^{*}$	0.0143${}^{*}$	0.0307${}^{*}$	0.0000	
DOS	0.0559${}^{*}$	0.0501${}^{*}$	0.1087${}^{*}$	0.1065${}^{*}$	0.0525${}^{*}$	0.0678${}^{*}$	0.0975${}^{*}$	0.057${}^{*}$	0.0000

${}^{*}$ Mean the significance p value (P<0.05) in variance analysis.

### 3.2 Genetic differentiation within and between sheep populations

In order to analyze the degree of differentiation within and between populations, F statistics were calculated in the nine sheep populations. The results are shown in Table 2. The $F_{\text{IT}}$ value of the 26 microsatellite loci varied between 0.0281 (MCM140) and 0.4481 (SRCRSP5) in the nine populations. The $F_{\text{IS}}$ value ranged from -0.0500 (SRCRSP9) to 0.1551 (TGLA53). In the pairwise-population $F_{\text{ST}}$ analysis, the greatest divergence was observed between BAM and DOP (0.1212) and between BAM and DST (0.1212) (Table 4 and Fig. 1). According to the chi-square test of $F_{\text{ST}}$, all populations showed significant divergence (P<0.05) from each other (Table 4). The overall $F_{\text{ST}}$ of each locus for nine sheep populations ranged from 0.0473 to 0.1414, with a mean of 0.079. The results indicated that the genetic variation between these populations reached 7.9 %.

**Table 5:** Nei's genetic distances (above the diagonal) and Nei's standard genetic distances (below the diagonal) for nine sheep populations.

Population	STH	HUS	SUF	DST	DOP	GMM	BAM	BAS	DOS
STH		0.0924	0.195	0.2149	0.1766	0.2119	0.2658	0.1761	0.1762
HUS	0.1239		0.2238	0.2316	0.1896	0.1651	0.1846	0.1544	0.1467
SUF	0.2826	0.3014		0.171	0.285	0.2724	0.3058	0.2892	0.3385
DST	0.2651	0.2889	0.2111		0.2288	0.23	0.3046	0.2509	0.2892
DOP	0.236	0.2396	0.1833	0.169		0.3178	0.3418	0.2964	0.1384
GMM	0.1614	0.196	0,2014	0,2017	0.2006		0.0755	0.0639	0.131
BAM	0.1749	0.2487	0.2171	0.2295	0.2081	0.0781		0.071	0.2922
BAS	0.1261	0.1715	0.2035	0.2094	0.1907	0.0434	0.0736		0.1903
DOS	0.1389	0.1623	0.2062	0.1999	0.1211	0.2253	0.152	0.1033	

**Figure 2:**
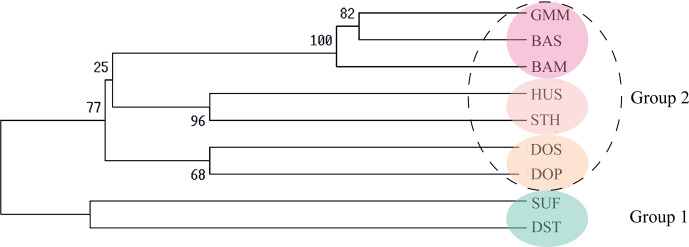
UPGMA phylogenetic tree of nine sheep populations based on Nei's genetic distance ($D_{\text{A}}$).

### 3.3 Phylogenetic relationship and population structure of nine sheep populations

Firstly, the genetic distance among nine sheep populations is shown in Table 5. The genetic distance $D_{\text{A}}$ ranged from 0.0434 to 0.3418 and $D_{\text{S}}$ ranged from 0.0639 to 0.2081. According to the Nei's genetic distance ($D_{\text{A}}$), an un-weighted pair-group method (UPGMA) phylogenetic tree of nine sheep populations was constructed (Fig. 2). The results indicated that nine sheep populations can be divided into two groups. SUF and DST were clustered in one group. The other seven populations were classed into another group. Then the second group was further divided into three clusters: GMM–BAS–BAM, HUS–STH and DOS–DOP.

**Figure 3:**
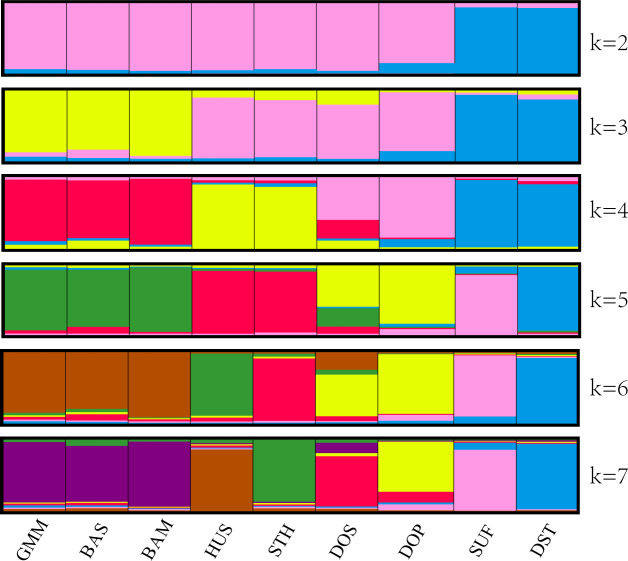
Population structure of nine sheep populations using model-based clustering method.

The above clustering results were also confirmed by structural analysis. In Fig. 3, each population is represented by a rectangle broken into k colored segments. At k=2, all the populations were separated into two different groups: the first group comprised SUF and DST, and the other consisted of the remaining seven populations. At k=3, the remaining seven populations were divided into two clusters (GMM–BAS–BAM and HUS–STH–DOS–DOP). At k=4, HUS and STH were clustered into one new subgroup, and DOS and DOP were clustered into another subgroup. In addition, from k=5 to k=7, no more new clusters generated. The clustering results were highly consistent with the UPGMA phylogenetic tree.

**Figure 4:**
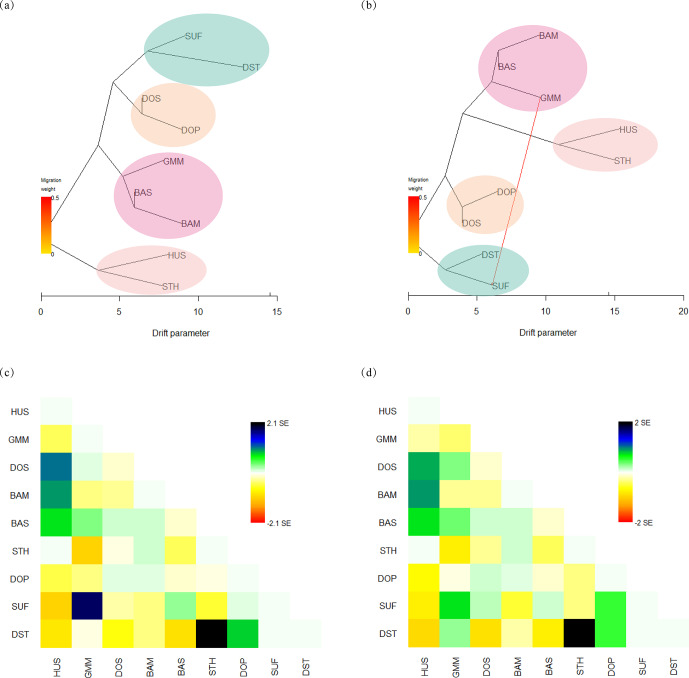
Maximum likelihood trees among nine sheep populations constructed with TreeMix. **(a)** Maximum likelihood tree without migration events; **(b)** maximum likelihood tree with one migration event; **(c)** residual fit without migration events; **(d)** residual fit with one migration event.

To infer migration events between populations, a maximum likelihood tree (Fig. 4a, b) was built using TreeMix. The residuals from the trees were inspected to estimate how well the model fit the data (Fig. 4c, d). Residuals above or below zero indicate that populations are more or less close to each other than they are presented in the tree. Based on this, it is implied that the actual genetic relationships between SUF and GMM and between DST and STH were closer than the evolutionary tree shows when no migration events were allowed (Fig. 4a, c). The model with one migration event (Fig. 4b) alluded an obvious gene flow from GMM to SUF. Combining the maximum likelihood tree (Fig. 4b) and residual plot (Fig. 4d), it was suggested that DST was more closely related to GMM than presented in the tree. However, when two or more migration events were assumed, the phylogenetic relationship shown in maximum likelihood tree was inconsistent with true breeding history of these sheep populations; therefore they are not shown in Fig. 4.

## 4 Discussion

The results of genetic variation analysis give us some hints. Firstly, overall, the nine sheep populations in this study have high genetic diversity and their mean $H_{\text{E}}$ and $H_{O}$ reached 0.71 and 0.68, respectively. The higher the genetic heterozygosity, the greater the variation of population (Bai et al., 2014; Dotsev et al., 2018; Jawasreh et al., 2018). Therefore, the large genetic variation existed within these investigated sheep populations. Secondly, as far as single breed is concerned, HUS has a higher genetic diversity, while STH did not display the expected diversity. This is consistent with the actual situation that HUS is being valued more in recent years, and therefore the number of being raised is very large, while the number of purebred STH is decreasing gradually. From the perspective of genetic diversity indicators ($H_{\text{E}}$ and $H_{\text{O}}$), the diversity of the current STH population is reduced compared to a few years ago (Ma et al., 2006; Liu et al., 2014). So, it is essential to formulate timely measures to protect genetic diversity of the famous prolific breed for meeting different needs in the future. In addition, the genetic diversity of four introduced mutton breeds (especially in DST) was relatively low in this study; therefore the result reminds us that the new individuals without kinship to current population should be added into these populations in order to keep rich genetic diversity and maintain excellent production performance as well as avoid inbreeding depression. Thirdly, for sheep in different regions of the world, Chinese sheep populations have an upper-middle level of genetic diversity, which is similar with previous reports (Niu et al., 2012; Liu et al., 2014; E et al., 2016, 2018). The genetic diversity in these Chinese sheep populations is higher than that of several breeds in Africa (e.g., West African Dwarf and Uda in Nigeria) (Agaviezor et al., 2012) and Europe (e.g., Cres island sheep and Lika pramenka sheep in Croatia; Denmark sheep in eastern Europe) (Tapio et al., 2010; Salamon et al., 2014). Compared with foreign sheep breeds, the intensity of artificial selection in Chinese sheep breeds is weak, which may be the reason for their high genetic diversity. Finally, the $F_{\text{ST}}$ among nine sheep populations (7.9 %) is similar to results of other studies in sheep (Dossybayev et al., 2019; Dudu et al., 2020), implying that a moderate genetic differentiation (5 %–15 %) exists among sheep populations.

The results of the UPGMA phylogenetic tree and structure analysis are consistent with breeding history of these sheep breeds. First of all, SUF and DST were clustered in one group. Since both SUF and DST horn were originally bred in the UK and both of their sire lines are Southdown rams (China National Commission of Animal Genetic Resources, 2011), genetic relationships between the two breeds were relatively closer and clustered together. Then the other seven sheep populations were clustered into another group. Of them, the three populations (GMM, BAM and BAS) were classed in one subgroup. The sire line in the initial breeding process of BAM and BAS is GMM and BAM rams, respectively. Therefore, a close genetic relationship existed among the three populations. Then, both HUS and STH belong to short fat-tailed Mongolian sheep (China National Commission of Animal Genetic Resources, 2011; Wang et al., 2014; Wei et al., 2015). A previous study suggested that, from the Mongolian Plateau, short fat-tailed sheep migrated southeast to the east of China (Zhao et al., 2017), which is a current distribution area of HUS and STH. Therefore, the two breeds were clustered together.

The gene flow and genetic exchange in the nine sheep populations were elucidated by TreeMix analysis. The results implied the possible gene flow from GMM to SUF. First of all, the gene flow is probably related to the history of human migration in Europe. From the 7th century BCE to the 3rd century BCE, Celtic tribes living in Germany had a great westward migration, and some of them entered Britain (Byrne et al., 2018). Then, from the fifth century CE, some of the Saxons and Angles of Germanic tribes again poured into Great Britain from Germany. Sheep has been considered as an important and easy-to-carry domestic animal for human several thousand years ago; therefore the gene flow between GMM and SUF might appear following human migrations, commercial trade, and extensive transport. Secondly, the gene flow was associated with the widespread use of Merino sires across Europe that commenced after the Middle Ages. Therefore, extensive haplotype sharing between Merino (including GMM) and other European breeds was observed (Kijas et al., 2012). Thirdly, previous analysis on the genetic relationships among world's sheep breeds suggests a major sheep migration route from South-West Asia to Britain and the Nordic regions, whose end potentially includes the route from Germany to the Britain (Kijas et al., 2012). Meanwhile, the genome-wide analysis of world's sheep breeds reveals frequent genetic exchange occurred during the development of modern sheep breeds (Kijas et al., 2012). In summary, these results can help us further understand the migration history and genetic relationship among populations for this important livestock species.

## 5 Conclusions

As a whole, these sheep populations in this study exhibited rich genetic diversities. However, specifically for individual breeds, STH and the four introduced populations did not display the expected diversity. The results of the UPGMA phylogenetic tree and the structure analysis are consistent with sheep breeding history. TreeMix analysis hinted at a possible gene flow from GMM to SUF. Together, these results will have important implications for sheep breed-specific management.

## Data Availability

The data sets are available upon request from the corresponding author.
